# Effectiveness of brief group intervention in the harmful alcohol use in primary health care

**DOI:** 10.11606/S1518-8787.2019053000498

**Published:** 2018-12-20

**Authors:** Janaina Soares, Divane de Vargas

**Affiliations:** I Escola de Enfermagem da Universidade Federal de Minas Gerais. Departamento de Enfermagem Aplicada. Belo Horizonte, MG, Brasil; II Escola de Enfermagem da Universidade de São Paulo. Departamento de Enfermagem Materno-Infantil e Psiquiátrica. São Paulo, SP, Brasil

**Keywords:** Alcoholism, Prevention & Control, Primary Care Nursing, Primary Health Care, Randomized Controlled Trial, Alcoolismo, prevenção & controle, Enfermagem de Atenção Primária, Atenção Primária à Saúde, Ensaio Clínico Controlado Aleatório

## Abstract

**OBJECTIVE:**

To verify the effectiveness of brief group intervention, performed by nurses, in reducing the hazardous or harmful alcohol use in users of a primary health care service.

**METHODS:**

Clinical and randomized trial with follow-up of three months. The sample had 180 individuals with a pattern of hazardous or harmful alcohol use, recruited in a Basic Health Unit in the city of São Paulo. A sociodemographic questionnaire and the Alcohol Use Disorders Identification Test (Audit) were applied. The experimental group underwent the Brief Group Intervention, which had four group sessions, with weekly meetings. The control group received an information leaflet about issues related to alcohol consumption. Both groups participated in the follow-up of three months. The linear mixed model was used for data analysis, in which a 5% significance level was adopted.

**RESULTS:**

Forty-four individuals under hazardous or harmful alcohol use completed all phases of the research. The experimental group had a statistically significant reduction (p < 0.01) of about 10 points in Audit score after the brief group intervention [before BGI = 15.89 (SD = 6.62) - hazardous use; after BGI = 6.40 (SD = 5.05) - low hazardous use] maintaining the low hazardous use in follow-up [6.69 (SD = 6.38) - low hazardous use]. The control group had a statistically significant reduction (p ≤ 0.01) of about three points in Audit score [before BGI = 13.11 (SD = 4.54) - hazardous use; after BGI = 9.83 (SD = 5.54) - hazardous use] and in follow-up presented the mean score of 13.00 (SD = 5.70), indicative of hazardous use. Differences between the two groups (experimental group versus control group) in reduction of consumption were statistically significant (p ≤ 0.01).

**CONCLUSIONS:**

Our evidence showed that the brief group intervention performed by the nurse in the primary health care context was effective to reduce alcohol consumption in individuals with patterns of hazardous or harmful use.

## RESUMO

**OBJETIVO:**

Verificar a efetividade da intervenção breve grupal realizada por enfermeiros, na redução do uso de risco e nocivo de álcool em usuários de um serviço de atenção primária à saúde.

**MÉTODOS:**

Ensaio clínico, randomizado, com*follow-up* de três meses. A amostra foi composta de 180 indivíduos que apresentaram padrão de uso de risco ou nocivo de álcool, recrutados em uma Unidade Básica de Saúde do município de São Paulo. Foi aplicado um questionário sociodemográfico e o *Alcohol Use Disorders Identification Test* (Audit). O grupo experimental foi submetido à Intervenção Breve Grupal, a qual foi composta por quatro sessões grupais, com encontros semanais. O grupo controle recebeu um folheto informativo sobre problemas relacionados ao consumo de álcool. Ambos os grupos participaram do seguimento de três meses. O modelo linear misto foi utilizado para análise dos dados, em que foi adotado nível de significância de 5%.

**RESULTADOS:**

Quarenta e quatro indivíduos que faziam uso de risco ou nocivo de álcool, completaram todas as fases da pesquisa. O grupo experimental apresentou redução estatisticamente significativa (p ≤ 0,01) de cerca de 10 pontos no escore do Audit após a intervenção breve grupal [antes IBG = 15,89 (dp = 6,62) - uso de risco; após IBG = 6,40 (dp = 5,05) - uso de baixo risco] mantendo o uso de baixo risco no seguimento [6,69 (dp = 6,38) - uso de baixo risco]. O grupo controle apresentou redução estatisticamente significativa (p ≤ 0,01) de cerca de três pontos no escore do Audit [antes = 13,11 (dp = 4,54) - uso de risco; após = 9,83 (dp = 5,54) - uso de risco] e no seguimento apresentou o escore médio de 13,00 (dp = 5,70) indicativo de uso de risco. As diferenças entre os dois grupos (grupo experimental *versus* grupo controle) na redução do consumo foram estatisticamente significativas (p ≤ 0,01).

**CONCLUSÕES:**

Evidenciou -se que a intervenção breve grupal realizada pelo enfermeiro no contexto da atenção primária à saúde foi efetiva para a redução do consumo de álcool em indivíduos com padrão de uso de risco ou nocivo.

## INTRODUCTION

Every year, the estimate is that about two billion people, approximately 40% (two out of every five people) of the world population over 15 years, consumes alcoholic drinks^1^. Of these, 16% makes a harmful use (alcohol use pattern characterized by increasing the risks of health or social damage in user)^2^, a phenomenon that has been currently constituted in one of the biggest and most costly public health problems around the world. According to the Global Status Report on Alcohol and Health 2011^3^, the world regions with the highest levels of alcohol consumption are still considered Europe and Americas.

In the latter, 20.9% of the population over 15 years have heavy episodic drinking occasions (five or more doses on a single occasion). In Brazil, a study conducted by *Vigilância de Fatores de Risco e Proteção para Doenças Crónicas por Inquérito Telefônico* (VIGITEL - Surveillance System of Risk and Protective Factors for Chronic Diseases by Telephone Survey), to investigate alcohol consumption among the adult population in the country, showed that the frequency of harmful consumption of alcoholic drinks in the past 30 days was 18.4%^4^.

Despite the morbidity and mortality of the harmful alcohol use, few studies have been conducted in Brazil about the prevalence of individuals with problems related to alcohol use in Primary Health Care (PHC) Units. These studies^5^ ^–^ ^8^ evidenced a prevalence ranging between 3% and 10% of people with problems related to alcohol use in these services. This suggests that a significant portion of the population attended in PHC services makes hazardous or harmful alcohol use. The PHC context is a privileged space for health prevention practice, since primary health care services are the first point of contact of individuals, families and communities in most countries^9^. For this reason, these spaces have been identified as strategic in the confrontation of several aggravations to population health, including the problematic alcohol use.

To face this particular issue, since 2001 the World Health Organization (WHO) has been recommending the implementation of brief interventions (BI)^10^, which has been proven effective in this context for reducing problematic alcohol use, including in Brasil^11^. Although BI are indicated as important feature in reducing harmful or hazardous alcohol consumption in the PHC scenario, there are several barriers that complicate its deployment in Brazilian PHC services, among which outstand the lack of human resources^12^, the professionals’ lack of time^13^ ^,^ ^14^ and the high demand of users in services^5^ ^–^ ^8^.

Thus, considering that nurses constitute a significant portion of professionals in health services, including in primary care, and that group interventions are already part of their activities in the PHC context and have been shown as an effective strategy in combating several aggravations to the population health, we assumed that group intervention can also be a valuable resource in care to people that make hazardous or harmful alcohol use. Therefore, this study aimed at verifying the effectiveness of brief group intervention performed by nurses in reducing the hazardous or harmful alcohol use in users of a primary health care service.

## METHODS

Randomized and controlled clinical trial^15^ with follow-up of three months, held in a *unidade básica de saúde* (UBS - basic health unit), located in the administrative district of downtown São Paulo. Individuals aged over 18 who sought the UBS between January and July 2015, regardless of the reason alleged for the search were invited to participate in the study.

The inclusion criteria were: be over 18 years, have availability to attend the brief group intervention (BGI) during the time and schedule determined, as well as participate in the follow-up (initial assessment, after one month, and after three months); know how to read and write; and receive score consistent with Zones II and III of the Alcohol Use Disorders Identification Test (Audit). Individuals who, at the time of collection, presented visible behavior changes, were intoxicated, or had no availability to receive the follow-up were excluded from the sample.

### Research Team

The research team consisted of four nurses from the *Núcleo de Estudos e Pesquisas em Enfermagem em Adições* - *álcool e outras drogas* (Study and Research Center of Nursing in Addictions - alcohol and other drugs) of the Universidade de São Paulo; all trained for the development of screening and the BGI.

### Sample Size

The sample calculation was estimated based on the pilot study (n = 10)^16^, obtained from an analysis of variance (ANOVA) model for repeated measures that was significant with 95% power and 5% significance level. An effect size of 0.43 was observed based on the pilot test. Thus, for the effect size to be significant with type I and II errors specified in this model, the minimum sample necessary was of 10 individuals. Assuming that 30% of individuals allocated into the intervention group would refuse to participate in the first phase of research and that there would be a 20% loss in the follow-up of 90 days (Friction)^15^, the minimum sample was increased to 20 individuals, 10 being allocated into control group, and 10 into experimental group.

### Data Collection Instruments

To identify the pattern of alcohol consumption, the Audit was applied. It consists of 10 questions that assess recent use of alcohol, dependency symptoms, and alcohol-related problems. Based on Audit scores, the user's pattern of alcohol consumption can be classified into four risk levels: zone I (0 to 7 points: low-hazardous use or abstinence); zone II (8 to 15 points: hazardous use); zone III (16 to 19 points: hazardous or harmful use); and zone IV (above 20 points: possible dependence). This instrument was validated in Brazil and presents good levels of sensitivity (87.8%) and specificity (81%) for detection of harmful alcohol use, with good performance in primary health care services^17^. In addition, in Brazilian validation, the Audit showed a satisfactory reliability (0.8) and ability to respond to changes in alcohol consumption^17^.

### Ethical Aspects

The study was approved by the Research Ethics Committee of the institution and other necessary authorities based on Resolution 466/12 of the Brazilian National Health Council, under Protocol 772,025.

### Screening

Individuals that scored zone I of the Audit during screening received an educational leaflet about problems related to alcohol use; those who scored zone IV, besides receiving the information material, were referred to specialized reference services of UBS. Participants who obtained scores in zones II or III of the Audit, i.e. identified as cases of hazardous or harmful alcohol use, and that met the inclusion criteria of the study, were invited to participate in the survey and were submitted to randomization.

### Randomization

The randomization of subjects was performed by the draw of two cards with the initials C for control group and E for experimental group. Individuals who drew the card with the letter C were allocated into the control group. They received an invitation with the scheduling of phone contacts for assessment of the pattern of alcohol use with dates and schedules. Those who drew the card with the letter E were assigned to the experimental group. They received an invitation with dates, schedules and location of intervention sessions. After randomization, a sequential number of the study was generated according to the screening order for identification of each subject in the research and his/her record in the study database.

### Control Group

From the nurses, members of the control group received the feedback of the score with proper clarification, an educational leaflet about the problems related to alcohol use^18^ and an invitation to two phone assessments, one after a month and another within three months, counted from the last phone call, to verify the pattern of alcohol consumption during these periods.

### Experimental Group

From the nurses, members of the experimental group received the feedback of the score with proper clarification, an educational leaflet about the problems related to the alcohol use and an invitation to participate in four brief group intervention sessions. These participants were divided into groups with at least five subjects that received the intervention in four weekly meetings.

### Brief Group Intervention (BGI)

The brief group intervention suggested in this study was based on the combination of two methodologies for reducing consumption of alcohol and other drugs: the brief individual intervention^19^ and the guided self-change (GSC) treatment^20^.

The BGI is an intervention conducted in a group format, coordinated by a nurse, which aims at the behavior change to reduce alcohol consumption in people who make hazardous or harmful alcohol use. The intervention was held in a room provided by the UBS where the study was carried out. It consisted of four sessions from 60 to 120 minutes, in which:

- 1^st^ Session - Reflecting on consumption (presentation of the members, consumption pattern feedback, advice, and acceptance of responsibility);
- 2^nd^ Session - Discussing new paths (discussion of the decisional balance, triggers of use, and guidance);
- 3^rd^ Session - Planning actions for change (discussion on the options menu - enjoyable activities and option for change plan);
- 4^th^ Session - Getting into action (development of new action options and plans, discussion about the possible opportunities to test the options for action plan for change, risk factors, protection and guidance).

At the end of the fourth session, the BGI was closed and the participants were reminded that they would be contacted through telephone and invited to return for the final individual interview after three months.

### Follow-up

To compare participants’ pattern of alcohol use, the follow-up assessment was conducted, in which the control group and the experimental group underwent initial assessment and other two follow-up assessments. The first was conducted shortly after the fourth BGI session (follow-up of one month) and the last, three months after the BGI (follow-up of three months), by individual interviews.

### Data Analysis

A descriptive analysis (mean and percentage) was made based on data collected from proportions and mixed effects model for longitudinal data analysis^21^, i.e. to check the pattern of alcohol use between the control and the experimental group in the three periods of time evaluated (initial assessment, after one month, and after three months). For all analyses, a 5% significance level was adopted.

## RESULTS

**Figure 1:**
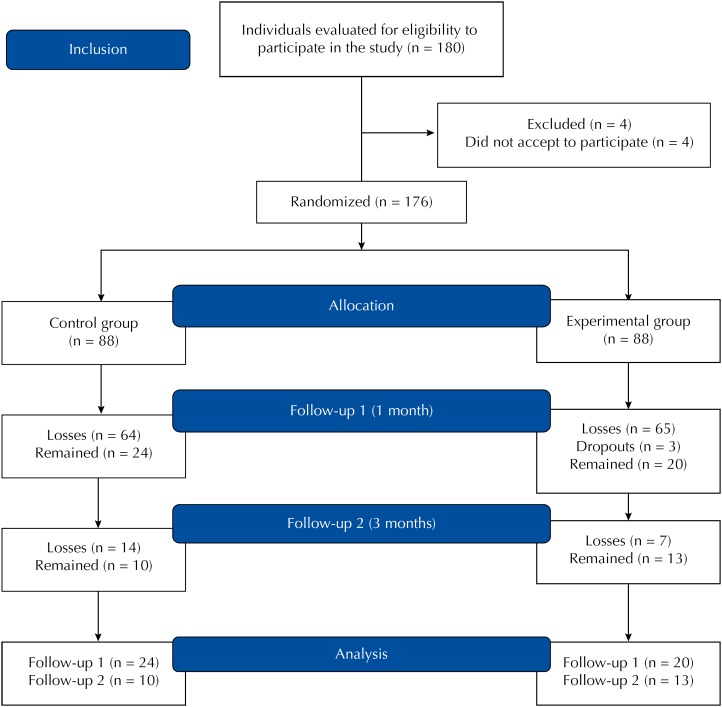
Flowchart of the sample of individuals participating in the study. São Paulo, 2015.

A total of 180 (20.7%) individuals with a hazardous or harmful pattern of alcohol use was tracked. Of these, four (2.2%) refused to participate, resulting in a potential sample of 176 (20.2%) individuals. Among the 176 possible participants, 88 (50%) were randomized to control group and 88 (50%) to experimental group. From the 176 potential participants, 44 were included in the final sample (Figure 1).

The control group consisted of 24 (27.3%) participants in the assessment after one month (follow-up 1). From these, 10 (41.6%) responded to the assessment after three months (follow-up 2). As for the experimental group, 23 participants attended the first BGI session; however, three withdrew, resulting in 20 (22.7%) participants that responded to the assessment after intervention (follow-up 1). Of these, 13 (65%) individuals responded to follow-up 2. Figure 1 illustrates the process of sample composition.

As for inferential analysis, the experimental group before intervention had a mean score of 15.89 (SD = 6.62) points (hazardous use). After intervention, the mean score was 6.40 (SD = 5.05) points (low hazardous use) and in the follow-up the mean score was 6.69 (SD = 6.38) points (low hazardous use). When scores of alcohol consumption between the three periods evaluated in the experimental group were compared, a statistically significant difference was noticed (p < 0.01), which remained in the follow-up.

**Figure 2:**
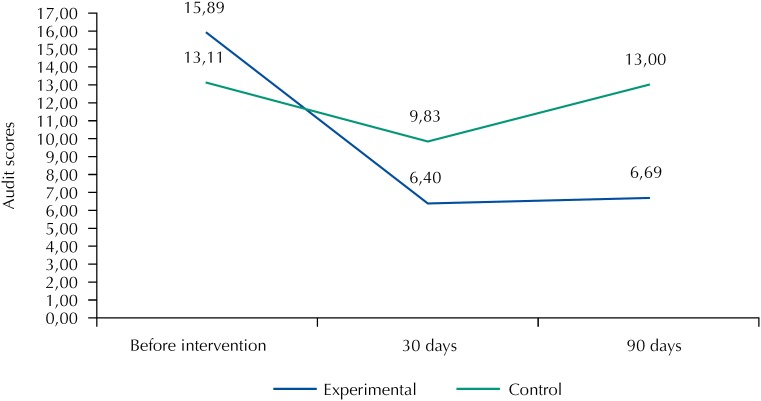
Score distribution of pattern of alcohol use observed between experimental and control groups according to the moment of assessment.

Considering the control group, the mean score in the first Audit assessment was 13.11 (SD = 4.54) points (hazardous use); in the second evaluation (after one month), the score had a mean of 9.83 (SD = 5.54) points (use); and in follow-up 2, the mean score was 13.00 (SD = 5.70) points (hazardous use), showing statistically significant difference in the Audit score after one month (p ≤ 0.01) (Figure 2).

Significant differences were found in the pattern of alcohol consumption between the experimental and control groups at baseline period versus after one month (p ≤ 0.01), basal period versus after three months (p = 0.03), and period after one month versus after three months (p = 0.01).

The experimental group had statistically significant difference (p ≤ 0.01) in the score for the pattern of alcohol consumption in about 10 points after intervention. The control group had a statistically significant difference of about three points (p ≤ 0.01) in the evaluation after one month. Regarding the follow-up, the experimental group maintained the decrease in score of alcohol consumption, as in the control group an increase of this score was noticed.

## DISCUSSION

This study was based on the construction of an intervention method in group format for reduction of alcohol use. This method saves time and human resources, through provision of care for a larger number of people in a single occasion.

The intervention proposed - participation in four BGI sessions - proved to be effective in reducing alcohol consumption of individuals with a pattern of hazardous or harmful alcohol use attending the UBS. This result is consistent with studies that endorse the group format in the confrontation of this problematic^22^ ^,^ ^23^.

The control group that received the information leaflet about alcohol consumption, despite having reduced the overall Audit score, remained with a compatible score with the hazardous risk throughout the follow-up period. This suggests that, after one month, the participants of this group decreased consumption, although they remained with the pattern of hazardous use throughout the follow-up, returning to initial consumption pattern after three months. There were no significant difference in the reduction of alcohol consumption in this group.

Data also showed a considerable withdrawal rate in BGI participation, as a portion of subjects randomized to the experimental group could not participate in the intervention because of the unavailability of time. Therefore, it is necessary to increase the supply of this intervention for more than one period of the day or to schedule alternative periods that are more attractive and suitable to the population demands.

In addition, one can infer that the implementation of the BGI practice by the UBS team itself is the most appropriate. Since the lack of interviewers’ link with the UBS population studied may have influenced the withdrawal rate, the implementation by UBS professionals could reduce the number of dropouts, given their greater bond with users of the service^24^ ^,^ ^25^. However, despite withdrawals, of all participants who initiated the BGI sessions, only 15% gave up (n = 3). This indicates that, when a person participates in the first BGI session, his/ her chances of participating in all sessions are high.

It is important to note that the participation of the population in interventions related to alcohol consumption is a challenge. To address the problematic alcohol use with the population is an unusual practice, which, along with the stigma and the moral weight of the issues related to alcohol consumption^26^, can cause resistance for people to accept participation in interventions. Moreover, to the common sense, only people with alcohol dependency must take care of this problem^27^.

This justifies the need of implementing practices related to hazardous or harmful alcohol use in the context of Brazilian PHC. Currently these services aim at preventing and reducing the damages of health risk behaviors. However, when it comes to the health care for alcohol users, there is a shortage of practices related to early interventions for the development of alcohol dependence in these health services^28^.

The implementation of interventions in PHC services must be included in the guidelines of the policies of the service. This would strengthen the importance of the dispensation of this practice in the service and would justify health professionals’ actions and involvement in the building of care protocols for application of effective interventions^12^ ^,^ ^29^.

This is the first study held in Brazil that addresses the BGI development conducted by nurses in primary health care services. It is consistent with the policy of comprehensive care to users of alcohol and other drugs^30^, which has among its objectives the prevention and reduction of damages, and directs the implementation of brief intervention practices in health services, especially those of PHC. On the other hand, the study results suggest that, despite being little used in research related to the intervention practice for alcohol users, the nurse was proven effective when trained to develop the BGI.

Finally, the BGI can be a resource to be considered in Brazilian health services because the format was proven to be an alternative and lower-cost strategy in relation to the individual brief interventions, given that, for their application, a lower number of human resources was required and a larger number of people were seen simultaneously. In addition, the practical deployment of BGI to health services can ensure them to comply with its objectives in reducing the alcoholism dependency rate and the long-term and expensive treatment, since BGI is a more viable practice to be employed in these services, considering its low cost and effectiveness.

Despite the evidence presented, the sample studied was minimal, which defines the importance of a replication of this study on a larger sample to allow a safer generalization of results. In addition, only one health service was used as study location; for a more robust analysis of the BGI practice, we suggest the conduction of multi-center studies to cover the practice of this technology in other health services.

This study contributes to the propagation of a low-cost intervention that can be deployed in the routine of health services. It prioritizes the early identification of the problem aiming at preventing future health complications to the population. This can avoid high-cost problems (long and chronic treatments). Additionally, the BGI can be replicable in different realities with regard to overall health.

Among the implications for research, this was a pioneering study that addressed the BGI effectiveness performed by the nurse to reduce alcohol consumption in PHC. For being the first study on a new form of using one technology of effective intervention, it becomes necessary to seek new evidence regarding the BGI use by other health professionals, as well as the practice of intervention performed by this professional in other specific populations and in other scenarios.

## CONCLUSION

The BGI, held by the professional nurse, is proven as a valid care strategy for reducing alcohol consumption in a population seen in an PHC service.

In view of the results of this study, we suggest that this technique is replicated in other Brazilian services, given that group interventions require less time of health professionals to see a larger number of patients than in the individual format.

However, despite BGI being an intervention of short duration (four sessions), perhaps it will be necessary to test BGI in a larger space of time between sessions, or even, in a lesser number of sessions to reduce the participants’ withdrawal, without losing the effectiveness.
